# Supplementary material for: A Likelihood Approach for Real-Time Calibration of Stochastic Compartmental Epidemic Models
Source: PLoS Comput Biol. 2017 Jan 17;13(1):e1005257. doi: 10.1371/journal.pcbi.1005257 (PMC5240920; doi:10.1371/journal.pcbi.1005257)
Supplement: S1 File — (TAR.GZ) [file pcbi.1005257.s014.tar.gz › HSPH_Online-SI-Revision/Additions/EnKF08/n100-extreme-SEITR_noisy/n100-extreme-SEITR_noisy_EnKF_table-4.pdf]

1	R0	{1.38654, 9.85565}	{2.69806, 4.1238}	{{2.16937, 1.77532}, {3., 6.10028}}	{94.5895, 58.158}	inf	4942.	6991.33	{839.396, 8650.2}	112.114	0.556118	newly	7087.2	11 129.3	{1385., 9865.}	171.302	0.52755	Reff	1.09645	0.531089	{0.229791, 0.677998}	62.659	ar	92 379.	200 313.	{183 761., 207 445.}	116.838
2	R0	{2.36629, 12.2029}	{2.0517, 4.33919}	{{1.01806, 2.6523}, {3., 4.99256}}	{36.4788, 64.4414}	inf	1894.	15 116.8	{4856.31, 58 889.4}	445.445	0.575201	newly	3034.	16 480.3	{6481., 57 252.}	446.096	0.556254	Reff	2.22479	0.36898	{0.037803, 1.12739}	83.7933	ar	99 022.	161 351.	{82 493., 238 876.}	70.696
3	R0	{2.32253, 12.836}	{2.5894, 7.47229}	{{2.18575, 4.18664}, {3., 14.8185}}	{17.511, 47.1703}	inf	1753.	21 952.2	{5734.86, 41 014.}	737.833	0.828616	newly	2684.	22 476.1	{4865., 52 037.}	743.431	0.798872	Reff	2.19238	1.00262	{0.311555, 2.18573}	54.4205	ar	98 997.	156 759.	{102 789., 225 577.}	62.8666
4	R0	{1.79075, 11.2058}	{2.26583, 4.03561}	{{1.14283, 2.64748}, {3., 4.93062}}	{52.3373, 63.9865}	inf	2803.	17 443.3	{9269.2, 56 791.9}	333.33	0.521408	newly	3933.	18 604.9	{7141., 55 602.}	376.522	0.534421	Reff	1.61667	0.334772	{0.0566986, 0.55349}	79.6232	ar	96 920.	162 180.	{81 327., 275 801.}	76.6429
5	R0	{2.26952, 9.62371}	{2.49664, 6.88957}	{{2.16325, 4.21693}, {3., 14.7617}}	{18.8777, 41.7005}	inf	1270.	23 513.4	{6813.13, 40 745.}	1091.41	0.963484	newly	3231.	26 413.9	{6793., 73 391.}	718.103	0.770569	Reff	2.17415	1.01557	{0.268232, 2.18633}	53.6682	ar	98 881.	154 241.	{78 129., 236 904.}	64.3937
6	R0	{1.57191, 6.89004}	{2.35412, 4.52051}	{{1.74308, 0.928216}, {3., 12.9972}}	{49.9683, 73.4788}	inf	1343.	27 005.4	{4747.08, 56 758.7}	1274.32	1.00183	newly	3690.	29 853.8	{4877., 69 448.}	710.37	0.770071	Reff	1.49482	0.560673	{0.203968, 1.29826}	63.9112	ar	95 046.	159 714.	{119 017., 248 319.}	72.0591
7	R0	{1.99226, 17.2339}	{2.28837, 5.19496}	{{0.863601, 0.833285}, {3., 18.2576}}	{48.8194, 72.2325}	inf	7484.	6852.55	{1062.31, 6344.15}	70.6915	0.517031	newly	6848.	9254.1	{1087., 12 258.}	96.0882	0.399693	Reff	1.39466	0.410272	{0.0899013, 0.612105}	72.7082	ar	97 992.	188 167.	{171 995., 232 908.}	92.0228
8	R0	{2.63369, 15.1879}	{2.18872, 4.05877}	{{1.08589, 2.65337}, {3., 4.94215}}	{30.8266, 73.2763}	inf	3695.	14 639.3	{6238.53, 34 421.7}	151.156	0.29393	newly	4729.	17 393.1	{6583., 51 176.}	273.611	0.422788	Reff	2.31741	0.409707	{0.0579624, 0.906685}	82.3205	ar	99 451.	166 337.	{75 398., 204 703.}	75.1972
9	R0	{2.53271, 9.90552}	{2.61488, 7.05199}	{{2.16336, 4.29401}, {3., 11.9581}}	{17.4199, 39.4848}	inf	823.	21 560.7	{6448.25, 40 738.4}	1609.1	1.09036	newly	2227.	23 635.3	{3997., 60 547.}	963.806	0.835486	Reff	2.46668	0.916299	{0.30616, 2.16369}	62.853	ar	99 355.	144 043.	{85 495., 219 691.}	53.2925
10	R0	{2.48791, 18.9003}	{2.58301, 4.97104}	{{1.995, 1.45184}, {3., 7.25588}}	{21.7006, 73.6986}	inf	5017.	3335.6	{822.002, 4785.32}	90.2124	0.80128	newly	4646.	3935.79	{732., 14 498.}	105.075	0.612384	Reff	2.05964	0.501638	{0.126267, 1.17907}	75.6444	ar	99 291.	180 663.	{171 574., 219 405.}	88.1423
11	R0	{2.84414, 18.4765}	{2.47074, 6.64323}	{{1.44733, 3.36326}, {3., 9.81174}}	{19.2663, 64.045}	inf	3971.	3140.26	{823.248, 7311.86}	82.0858	0.585355	newly	4035.	4213.11	{496., 7377.}	118.106	0.544054	Reff	2.47881	0.501127	{0.10777, 1.04097}	79.7836	ar	99 654.	193 150.	{181 190., 235 285.}	93.8207
12	R0	{2.29369, 14.622}	{2.50709, 4.7101}	{{1.99207, 1.44732}, {3., 7.20634}}	{22.7215, 67.7876}	inf	4819.	1717.41	{646.649, 2576.32}	82.0828	0.817781	newly	5911.	2922.93	{684., 4829.}	79.2873	0.671138	Reff	1.91186	0.556088	{0.150226, 1.19084}	71.6688	ar	98 982.	187 046.	{171 756., 237 758.}	90.7205
13	R0	{2.73413, 16.7529}	{2.15542, 4.6347}	{{0.0924215, 0.929515}, {3., 5.71747}}	{33.224, 72.3349}	inf	4263.	5345.45	{386.363, 13 064.5}	71.819	0.462252	newly	4789.	5514.6	{356., 13 436.}	83.6788	0.420567	Reff	2.35852	0.330445	{0.0136471, 0.450354}	85.9893	ar	99 543.	179 881.	{164 291., 213 641.}	81.5949
14	R0	{1.79554, 16.5943}	{2.74284, 3.93043}	{{2.29788, 1.8674}, {3., 6.10674}}	{52.7587, 76.3146}	inf	6015.	5046.5	{636.142, 7678.22}	92.0555	0.74342	newly	5421.	8185.29	{894., 10 557.}	163.267	0.571333	Reff	1.3756	0.435028	{0.0669213, 0.715048}	75.6463	ar	96 821.	180 190.	{164 757., 217 973.}	86.1058
15	R0	{1.91429, 13.7771}	{2.48673, 5.94237}	{{0.850061, 0.733094}, {3., 18.3879}}	{52.1386, 72.9304}	inf	5411.	8306.73	{722.847, 6717.3}	76.9178	0.392927	newly	6031.	9602.01	{811., 13 312.}	119.257	0.493744	Reff	1.53354	0.456771	{0.0239861, 0.638036}	70.2146	ar	97 541.	193 779.	{177 876., 192 899.}	98.6639
16	R0	{1.42094, 10.5871}	{2.28319, 8.1229}	{{0.317009, 2.8226}, {3., 12.8251}}	{88.6401, 41.7257}	inf	3733.	3789.62	{83.0799, 3421.77}	76.4435	0.654352	newly	4920.	3510.25	{71., 7228.}	85.6262	0.750115	Reff	1.20628	0.423339	{0.0658864, 0.655085}	65.1356	ar	93 044.	177 507.	{169 039., 198 153.}	90.7775
17	R0	{1.45515, 6.16466}	{2.48527, 6.8784}	{{2.16421, 4.17158}, {3., 14.8202}}	{73.7392, 48.3328}	inf	733.	21 983.3	{5834.47, 38 940.5}	2155.03	1.19513	newly	2188.	26 128.8	{4360., 72 037.}	1098.36	0.880101	Reff	1.41733	1.03262	{0.35446, 2.097}	49.2521	ar	93 254.	154 948.	{73 098., 238 320.}	73.639
18	R0	{2.85669, 13.2416}	{2.3692, 5.16041}	{{1.74774, 0.993776}, {3., 13.0515}}	{21.5797, 61.9965}	inf	1540.	22 539.9	{4641.1, 53 467.6}	814.632	0.834163	newly	2807.	27 117.4	{4069., 69 068.}	872.6	0.822998	Reff	2.71777	0.615085	{0.141695, 1.30054}	77.368	ar	99 644.	159 891.	{78 021., 250 308.}	69.0601
19	R0	{2.56417, 17.9558}	{2.2582, 8.64738}	{{0.391667, 2.87684}, {3., 12.9974}}	{29.6093, 51.8407}	inf	4153.	3421.21	{82.7892, 2647.14}	79.8872	0.648257	newly	4054.	2575.4	{40., 3914.}	72.0064	0.596004	Reff	2.21442	0.384666	{0.0614857, 0.586588}	82.629	ar	99 389.	185 031.	{177 921., 183 988.}	86.1684
20	R0	{2.27517, 11.1864}	{2.41455, 4.09687}	{{1.7694, 1.05679}, {3., 12.7539}}	{24.7226, 67.0983}	inf	2051.	24 971.6	{4928.92, 54 299.2}	718.157	0.797	newly	3692.	31 304.3	{4925., 76 607.}	755.532	0.786245	Reff	2.12451	0.609563	{0.243321, 1.35995}	71.3081	ar	98 894.	164 811.	{107 852., 256 846.}	73.0009
21	R0	{2.35735, 10.4735}	{2.62348, 7.15946}	{{2.1743, 4.18041}, {3., 14.7474}}	{19.7891, 42.235}	inf	1087.	22 387.3	{1230.38, 55 174.2}	1297.49	0.967312	newly	2417.	23 705.9	{1459., 61 191.}	892.61	0.81321	Reff	2.2751	0.985098	{0.445258, 2.18283}	56.7009	ar	99 036.	154 006.	{66 635., 232 418.}	66.1924
22	R0	{1.79551, 11.9179}	{2.5629, 5.0836}	{{2.00524, 1.43152}, {3., 7.18895}}	{44.0637, 57.3448}	inf	4439.	1479.12	{761.026, 2434.28}	79.1163	0.750137	newly	5958.	2917.53	{738., 4533.}	92.4154	0.715316	Reff	1.5037	0.493595	{0.162029, 1.34603}	73.2335	ar	97 033.	187 357.	{174 065., 222 364.}	93.0853
23	R0	{1.23545, 6.31687}	{1.82194, 4.35977}	{{0.0307622, 0.926815}, {3., 5.7034}}	{111.737, 38.8197}	inf	2814.	5080.03	{784.345, 18 365.1}	77.6081	0.316206	newly	6598.	5258.34	{684., 17 771.}	76.0164	0.48627	Reff	1.0892	0.279723	{0.00421905, 0.4622}	76.336	ar	88 739.	182 125.	{167 207., 236 764.}	106.076
24	R0	{2.24089, 10.1789}	{2.37035, 4.69759}	{{1.75238, 1.39055}, {3., 12.5938}}	{26.5909, 62.3989}	inf	976.	26 243.7	{4826.81, 60 702.2}	1780.35	1.07994	newly	2027.	28 549.	{4896., 74 098.}	1310.4	0.960661	Reff	2.17337	0.569647	{0.14696, 1.30389}	73.7897	ar	98 868.	160 101.	{74 341., 251 034.}	70.1575
25	R0	{1.57298, 10.2607}	{2.35368, 6.35593}	{{1.44023, 3.28099}, {3., 9.66842}}	{54.6459, 38.0556}	inf	2641.	2815.03	{229.108, 10 526.4}	100.298	0.671808	newly	3941.	4421.3	{166., 10 861.}	142.204	0.749945	Reff	1.41523	0.618206	{0.226828, 1.20875}	59.1372	ar	94 957.	184 356.	{169 205., 249 119.}	94.1467
26	R0	{1.2154, 8.2576}	{2.41661, 11.5588}	{{1.21419, 4.81818}, {3., 17.4062}}	{99.0766, 57.4635}	inf	4010.	6157.45	{1347.62, 13 529.6}	109.492	0.474243	newly	6408.	5988.58	{629., 13 587.}	131.992	0.740491	Reff	0.993966	0.608392	{0.169205, 1.42859}	62.7833	ar	88 421.	202 207.	{180 149., 283 076.}	128.686
27	R0	{2.22841, 18.7983}	{2.47139, 12.2174}	{{1.3862, 4.84659}, {3., 17.2993}}	{27.6363, 35.0078}	inf	7582.	4150.51	{762.403, 4154.04}	86.7473	0.829458	newly	6511.	4641.11	{500., 5400.}	119.941	0.821954	Reff	1.58429	0.677381	{0.0452832, 1.90067}	65.4742	ar	98 756.	189 932.	{170 999., 253 823.}	92.3241
28	R0	{1.27778, 5.36767}	{2.55843, 7.98209}	{{2.16483, 4.15256}, {3., 14.6818}}	{101.182, 63.0839}	inf	966.	20 814.5	{6934.7, 35 457.5}	1561.21	1.10783	newly	2971.	22 296.9	{5986., 58 862.}	653.412	0.7165	Reff	1.23118	1.01231	{0.357413, 2.18625}	48.9379	ar	89 843.	152 879.	{90 951., 219 566.}	73.5447
29	R0	{2.38042, 15.8901}	{2.05876, 4.28302}	{{0.0910224, 0.951537}, {3., 5.69553}}	{40.5692, 73.046}	inf	3337.	5797.91	{723.314, 18 858.5}	88.4488	0.436807	newly	3642.	6224.17	{597., 17 252.}	131.127	0.457497	Reff	2.11469	0.30179	{0.0122836, 0.433006}	86.2649	ar	99 112.	155 147.	{88 886., 220 419.}	64.7796
30	R0	{2.53188, 12.7607}	{2.51427, 7.45238}	{{2.1641, 4.29035}, {3., 14.6761}}	{16.2265, 46.7549}	inf	1016.	21 982.9	{6154.78, 39 861.9}	1459.43	1.08679	newly	1649.	25 842.2	{4367., 72 390.}	1468.61	1.00074	Reff	2.45506	1.00236	{0.519118, 2.18588}	59.1716	ar	99 317.	162 976.	{113 584., 241 689.}	66.9968
31	R0	{1.24482, 10.5471}	{2.71192, 4.24633}	{{2.25133, 1.57728}, {3., 6.13425}}	{117.856, 59.7394}	inf	5483.	7716.35	{830.759, 8688.39}	113.66	0.588714	newly	6713.	12 286.9	{1379., 22 800.}	187.192	0.530179	Reff	0.914831	0.565698	{0.187471, 0.890593}	53.4445	ar	89 097.	200 301.	{181 182., 252 254.}	124.812
32	R0	{2.30446, 17.9607}	{2.45851, 4.61507}	{{1.99438, 1.09325}, {3., 7.42929}}	{21.6864, 74.3046}	inf	5816.	2161.12	{677.704, 3091.18}	84.1132	0.904832	newly	5244.	3102.98	{666., 4583.}	84.585	0.630582	Reff	1.82382	0.548842	{0.158521, 1.20322}	71.4031	ar	99 028.	186 965.	{169 472., 246 638.}	88.8006
33	R0	{2.31579, 11.8236}	{2.52487, 6.90697}	{{2.17727, 4.10921}, {3., 9.71059}}	{18.867, 47.2024}	inf	3092.	21 718.7	{3671.24, 42 561.2}	374.251	0.592583	newly	5187.	21 976.	{4622., 53 400.}	336.197	0.53873	Reff	2.08296	0.915088	{0.27601, 2.15489}	57.4684	ar	99 012.	151 435.	{71 373., 232 806.}	61.6127
34	R0	{1.6678, 10.6744}	{2.50273, 4.5183}	{{1.11414, 1.42392}, {3., 7.20664}}	{56.8224, 57.6716}	inf	4713.	1730.35	{895.321, 2618.41}	83.3912	0.806294	newly	6918.	2120.25	{710., 4396.}	78.1363	0.749928	Reff	1.37288	0.507736	{0.147604, 1.11413}	70.8927	ar	95 809.	188 929.	{177 133., 229 859.}	98.8144
35	R0	{1.65476, 7.07559}	{2.47225, 6.46331}	{{2.1583, 4.23857}, {3., 9.9955}}	{54.615, 37.6535}	inf	893.	19 481.4	{5357.65, 34 149.7}	1456.48	1.12855	newly	2514.	23 945.1	{4742., 60 355.}	858.588	0.871465	Reff	1.60409	1.00394	{0.23971, 2.18495}	52.5351	ar	95 746.	145 052.	{67 742., 214 463.}	61.2104
36	R0	{2.71807, 18.5959}	{2.58668, 5.01861}	{{2.00358, 1.55798}, {3., 7.34053}}	{17.281, 73.0123}	inf	3397.	1431.13	{848.873, 2734.72}	73.0661	0.660823	newly	3163.	2126.52	{700., 4167.}	79.5599	0.504896	Reff	2.4286	0.478208	{0.176065, 1.13617}	80.3093	ar	99 540.	175 063.	{168 358., 200 635.}	80.1191
37	R0	{2.6202, 12.7552}	{1.80515, 4.44473}	{{0.0674801, 0.936581}, {3., 5.72152}}	{44.7316, 66.4494}	inf	1248.	5274.46	{518.267, 20 080.7}	217.992	0.42854	newly	2178.	6015.06	{489., 18 559.}	219.94	0.449658	Reff	2.51914	0.299048	{0.0046602, 0.517825}	88.129	ar	99 426.	171 675.	{144 597., 237 678.}	78.17
38	R0	{1.47672, 9.81651}	{2.12014, 9.42033}	{{0.297654, 2.51278}, {3., 13.6734}}	{81.8452, 44.5205}	inf	4210.	4190.48	{130.567, 3945.14}	111.731	0.941582	newly	6266.	3336.49	{62., 15 382.}	104.482	1.09633	Reff	1.23	0.414343	{0.0543222, 0.676862}	69.1871	ar	93 815.	174 511.	{163 403., 211 545.}	86.0158
39	R0	{1.90004, 12.3313}	{2.47508, 4.61212}	{{1.24032, 1.01819}, {3., 7.52251}}	{37.3447, 62.5982}	inf	3615.	1355.26	{603.826, 1971.9}	76.7409	0.716985	newly	4980.	1741.33	{729., 2878.}	75.9564	0.675595	Reff	1.65284	0.428952	{0.145978, 0.545004}	76.0401	ar	97 413.	186 215.	{178 001., 208 100.}	92.7178
40	R0	{2.64218, 18.5237}	{1.96611, 4.13163}	{{0.0401665, 0.936873}, {3., 5.66565}}	{39.9705, 77.6954}	inf	6412.	5013.54	{643.84, 15 998.4}	73.1607	0.622	newly	6088.	5439.08	{574., 13 470.}	75.7401	0.455842	Reff	2.0572	0.306771	{0.00594568, 0.455}	85.0879	ar	99 474.	163 192.	{144 962., 205 067.}	71.7517
41	R0	{1.6945, 7.17996}	{2.53565, 7.1603}	{{2.16305, 4.19544}, {3., 14.7294}}	{51.8502, 45.1548}	inf	498.	26 696.7	{7065.81, 66 211.6}	3996.11	1.46692	newly	1333.	29 236.2	{5783., 70 869.}	2095.	1.15593	Reff	1.66739	1.01509	{0.320871, 2.16516}	50.9435	ar	96 332.	152 297.	{78 705., 227 847.}	66.7703
42	R0	{1.84559, 18.0074}	{2.4095, 6.7223}	{{0.838563, 0.81298}, {3., 18.164}}	{55.6573, 63.1806}	inf	7319.	7975.65	{243.018, 6445.72}	78.9164	0.610345	newly	6151.	8285.7	{169., 12 223.}	96.6032	0.557106	Reff	1.29117	0.465728	{0.181589, 0.648502}	66.9994	ar	97 358.	190 063.	{172 683., 230 731.}	95.2205
43	R0	{2.24965, 17.0361}	{2.41139, 4.88803}	{{1.13367, 1.53429}, {3., 7.25372}}	{25.8611, 71.3078}	inf	4414.	2366.69	{944.5, 3054.41}	85.3887	0.767163	newly	4281.	3061.84	{678., 4547.}	89.2189	0.57321	Reff	1.90728	0.49993	{0.106608, 1.11486}	74.4021	ar	98 770.	187 573.	{172 119., 243 188.}	89.909
44	R0	{2.54324, 15.7967}	{2.54085, 4.53863}	{{1.99297, 1.51831}, {3., 7.22745}}	{20.2089, 71.2685}	inf	4842.	2581.18	{922.09, 2789.56}	90.2748	0.802058	newly	5449.	3170.04	{744., 4426.}	93.1965	0.674749	Reff	2.13836	0.446186	{0.106242, 0.999656}	79.1342	ar	99 354.	191 153.	{177 499., 209 320.}	92.3961
45	R0	{2.54943, 14.8032}	{2.06148, 4.1387}	{{1.04322, 2.64665}, {3., 4.92255}}	{35.112, 72.0419}	inf	3565.	14 829.5	{8424.64, 33 180.7}	168.382	0.339296	newly	4552.	16 433.7	{6545., 38 739.}	264.995	0.432411	Reff	2.25387	0.346695	{0.0678923, 0.852359}	84.6178	ar	99 380.	164 517.	{84 283., 199 362.}	72.6645
46	R0	{2.37755, 12.6461}	{2.53711, 7.38297}	{{2.16649, 4.08182}, {3., 14.7345}}	{16.6945, 45.2622}	inf	1629.	22 976.3	{6753.4, 49 352.7}	792.452	0.83897	newly	2717.	24 457.4	{6052., 60 022.}	806.075	0.798665	Reff	2.25026	0.927105	{0.309212, 2.09564}	58.8987	ar	99 096.	154 747.	{108 064., 221 605.}	58.9202
47	R0	{1.69279, 11.6636}	{2.45322, 6.5388}	{{1.41358, 3.31811}, {3., 9.4957}}	{52.8217, 43.9384}	inf	3683.	2717.96	{83.7151, 5082.63}	89.3209	0.769533	newly	4864.	3571.54	{48., 9032.}	110.216	0.822287	Reff	1.4668	0.518373	{0.0870331, 1.08673}	65.5525	ar	96 214.	182 997.	{168 361., 221 516.}	90.1977
48	R0	{1.82402, 10.0038}	{2.20601, 3.98643}	{{1.13528, 2.65565}, {3., 4.94179}}	{47.8568, 60.1508}	inf	1751.	17 415.5	{5110.4, 57 539.6}	575.872	0.70363	newly	3183.	18 610.2	{5622., 55 871.}	492.201	0.639448	Reff	1.71321	0.352399	{0.0503179, 0.78667}	79.4305	ar	97 076.	164 453.	{83 290., 215 839.}	76.5565
49	R0	{1.6424, 11.1667}	{2.43996, 5.05966}	{{1.98931, 1.28605}, {3., 7.1052}}	{51.4411, 54.6897}	inf	5534.	1467.59	{566.063, 2182.47}	83.4526	0.912222	newly	7611.	1871.26	{514., 4484.}	78.8785	0.85046	Reff	1.28365	0.49313	{0.139912, 1.01304}	68.1803	ar	95 546.	177 143.	{168 100., 199 437.}	88.8123
50	R0	{2.05638, 14.3048}	{2.0079, 4.4634}	{{0.052543, 0.936145}, {3., 5.66992}}	{51.9789, 69.1512}	inf	4454.	6607.87	{560.539, 18 621.6}	89.0839	0.488759	newly	5024.	8339.7	{484., 17 043.}	138.659	0.46643	Reff	1.73898	0.285181	{0.00794553, 0.444278}	83.6006	ar	98 194.	169 075.	{84 783., 201 217.}	82.8682
